# Development of the Platysma Muscle and the Superficial Musculoaponeurotic System (Human Specimens at 8–17 Weeks of Development)

**DOI:** 10.1155/2013/716962

**Published:** 2013-12-12

**Authors:** C. De la Cuadra-Blanco, M. D. Peces-Peña, L. O. Carvallo-de Moraes, M. E. Herrera-Lara, J. R. Mérida-Velasco

**Affiliations:** ^1^Departamento de Anatomía y Embriología Humana II, Facultad de Medicina, Universidad Complutense de Madrid, 28040 Madrid, Spain; ^2^Departamento de Anatomía y Embriología Humana I, Facultad de Medicina, Universidad Complutense de Madrid, 28040 Madrid, Spain; ^3^Department of Orofacial Sciences and Pediatrics, Program in Craniofacial and Mesenchymal Biology, University of California, San Francisco, CA 94143-0430, USA

## Abstract

There is controversy regarding the description of the different regions of the face of the superficial musculoaponeurotic system (SMAS) and its relationship with the superficial mimetic muscles. The purpose of this study is to analyze the development of the platysma muscle and the SMAS in human specimens at 8–17 weeks of development using an optical microscope. Furthermore, we propose to study the relationship of the anlage of the SMAS and the neighbouring superficial mimetic muscles. The facial musculature derives from the mesenchyme of the second arch and migrates towards the different regions of the face while forming premuscular laminae. During the 8th week of development, the cervical, infraorbital, mandibular, and temporal laminae are observed to be on the same plane. The platysma muscle derives from the cervical lamina and its mandibular extension enclosing the lower part of the parotid region and the cheek, while the SMAS derives from the upper region. During the period of development analyzed in this study, we have observed no continuity between the anlage of the SMAS and that of the superficial layer of the temporal fascia and the zygomaticus major muscle. Nor have we observed any structure similar to the SMAS in the labial region.

## 1. Introduction

Mitz and Peyronie [1] described the superficial musculoaponeurotic system (SMAS) in the parotid and cheek regions in human adults. Their description affirmed that the SMAS was located between the dermis and the facial muscles and divided the hypodermic fat into two layers. Superficial to the SMAS, small fat lobules were enclosed by fibrous septa running from the SMAS towards the dermis. Deep to the SMAS, the fat was abundant, lying between deep facial muscles and not divided by such fibrous septa.

The term SMAS is generally accepted in scientific literature. It is not included in anatomical terminology [2] although it is referred to in some human anatomy textbooks [3]. Surgical dissection of the SMAS, its mobilization, and traction reflect its functional importance in aesthetic plastic surgery [4–10].

Macroscopic and microscopic studies have been performed on the neck of adults and other facial regions to demonstrate the existence and structure of the SMAS and clarify its functional role [4, 11–15]. Radiological studies have also been performed to determine the morphology of the hypodermic tissue of the face, especially the SMAS [16].

There are, however, discrepancies regarding its morphological definition. Ghassemi et al. [14] assumed that the SMAS was a continuous, organized meshwork connecting the facial muscles with the dermis and was formed by a three-dimensional meshwork of collagenous, elastic fibres, fat cells, and muscular fibres. These authors distinguished two morphological types of SMAS; type 1 was located lateral to the nasolabial fold with relatively small fibrous septa enclosing lobules of fat cells, whereas type 2 was located medial to the nasolabial fold where the SMAS was composed of a dense collagen muscle fibre meshwork.

In Gray's Anatomy [3], the SMAS is described as a single plane in the face composed of muscular fibres in some areas and elsewhere of fibrous or fibroaponeurotic tissue not directly attached to bone. In Macchi et al. [15], the SMAS is described as corresponding to the superficial fascia in the face, functioning as a central tendon for coordinated contraction of the mimic musculature of the face.

In adults, the description of the SMAS in the different regions of the face has also been a subject of controversy. For some authors, the SMAS does not exist as an autonomous entity and may only be defined in the parotid region [17, 18]. In this region, the SMAS was different from the parotid fascia, being thick [1, 18] or thin [4, 12, 14, 19] and, moreover, separated from the parotid fascia by a thin lamina of deep adipose tissue [15].

Pensler et al. [11] considered that the SMAS was thin in the nasolabial fold and could be identified in the labial region while other authors believe that it does not reach the labial region [16, 18].

The superficial layer of the temporal fascia [2] corresponds to the temporal superficial layer or the temporoparietal fascia [3, 4, 6, 20]. In the temporal region, the SMAS is continuous and is on the same plane as the superficial layer of the temporal fascia [15, 20–22]. However, other authors consider that these structures are not continuous [3, 18, 19].

The relationship of the SMAS and the mimetic muscles presents notable discrepancies. Some authors consider that the SMAS encloses the mimetic muscles [15], while others affirm that the SMAS finalizes in the zygomaticus major muscle [9]. Other authors state that it is continuous with other mimetic muscles such as the zygomaticus major muscle, the frontal belly of the epicranius muscle, and the orbicularis oculi muscle [1, 3].

Although there appears to be a clear relationship between the SMAS and the mimic muscles, studies into its development are relatively few. Gasser's embryological studies [23] described and illustrated that the superficial mimetic musculature developed from common premuscle condensations. This author described the cervical, mandibular, infraorbital, temporal, and occipital laminae. The cervical and mandibular laminae in 20–23 mm greatest length (GL) specimens were united by a continuous, thick layer. At 26 mm (GL), the continuity between the cervical, mandibular, and infraorbital laminae was described. The superficial muscles differentiated rapidly between 26 and 37 mm (GL) and the plastysma muscle developed from the cervical lamina and its mandibular extension. Zigiotti et al. [24] in their studies on 10 human fetuses described the continuity of the platysma muscle with the superficial fascia in the parotid region. Gardetto et al. [18] analyzed the development of the hypodermic layers of the face and neck of 22 human fetuses and 3 newborns, concluding that the SMAS could only be described in the parotid region and was continuous with the platysma muscle.

Our objective has been to determine the origin and arrangement of the platysma muscle and the SMAS in human specimens at 8–17 weeks of development and also the relationship of the superficial mimetic muscles with the SMAS.

## 2. Materials and Methods

The study was performed in accordance with the provisions of the Declaration of Helsinki 1995 (revised in Edinburgh 2000). The regions of the face were investigated in human embryos and human fetuses belonging to the Embryology Institute of the Complutense University of Madrid. In the embryos, the greatest length (GL) ranged from 22 to 30 mm (4 of Carnegie stage 22 and 6 of 23), while in the fetuses, the GL ranged from 35 to 150 mm (5 of week 9, 4 of week 10, 6 of week 11, 5 of week 12, 4 of week 13, 3 of week 14, and 1 of each of weeks 15 and 17). The parameters used to determine the postconception age were the GL and external and internal criteria [25, 26]. All specimens were from ectopic pregnancies or spontaneous abortions and there were no indications of possible malformation. The sections, which included three spatial planes, were stained with haematoxylin-eosin (HE), azocarmine, Bielchowsky and Masson's trichrome dye [27].

The study was approved by the Ethics Committee of the Faculty of Medicine of the University Complutense of Madrid. For this study, the Terminologia Anatomica (1998) has been used.

## 3. Results

**Figure 1:**
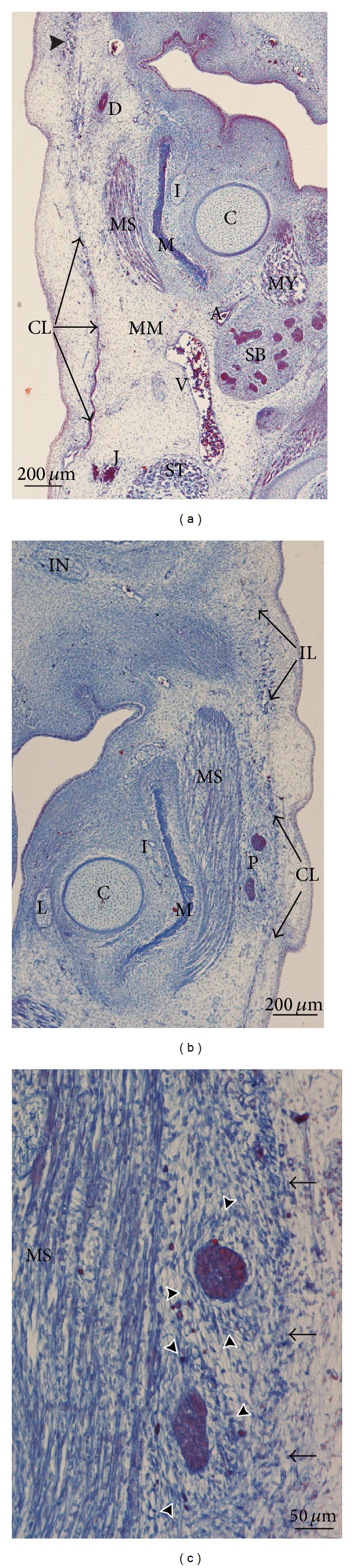
(a) Human embryo GI4 (26.5 mm GL; 8 weeks of development). Frontal section. Azocarmine staining. The cervical lamina (CL) extends towards the mandibular region enclosing the masseter muscle (MS). Arrowhead: infraorbital lamina. Bar: 200 *μ*m. (b) Human embryo GI4 (26.5 mm GL; 8 weeks of development). Frontal section. Azocarmine staining. The mandibular extension of the cervical lamina (CL) and the infraorbital lamina (IL) are visible. Bar: 200 *μ*m. (c) Magnification of (b). The parotid acini are surrounded by condensed mesenchyme, anlage of the capsula propia of the parotid gland (arrowheads). Arrows: mandibular extension of the cervical lamina. Bar: 50 *μ*m.

**Figure 2:**
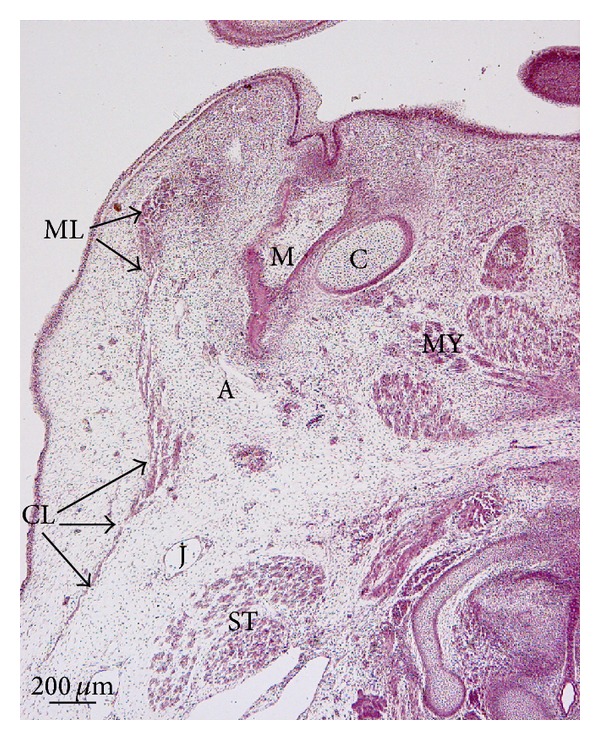
Human embryo Ca2 (29 mm GL; 8 weeks of development). Frontal section. Haematoxylin-eosin staining. The cervical lamina (CL) is continuous with the mandibular lamina (ML). Bar: 200 *μ*m.

In embryos at 8 weeks of development, the cervical, mandibular, infraorbital, and temporal laminae can be identified on the same plane. The cervical lamina encloses the sternocleidomastoid muscle, the external jugular vein, the submandibular gland the facial vessels, and the marginal mandibular branch of the facial nerve (Figure 1(a)). The mandibular extension of the cervical lamina encloses the parotid region and the masseter muscle. The parotid gland is made up of epithelial cords, the anlage of the parenchyma, enclosed by condensed mesenchyme, the anlage of the capsule propia (Figures 1(b) and 1(c)). In the area of the lower lip, the continuity between the cervical and mandibular laminae can be observed (Figure 2). We have observed no continuity between the mandibular extension of the cervical lamina and the infraorbital lamina (Figures 1(a) and 1(b)).

**Figure 3:**
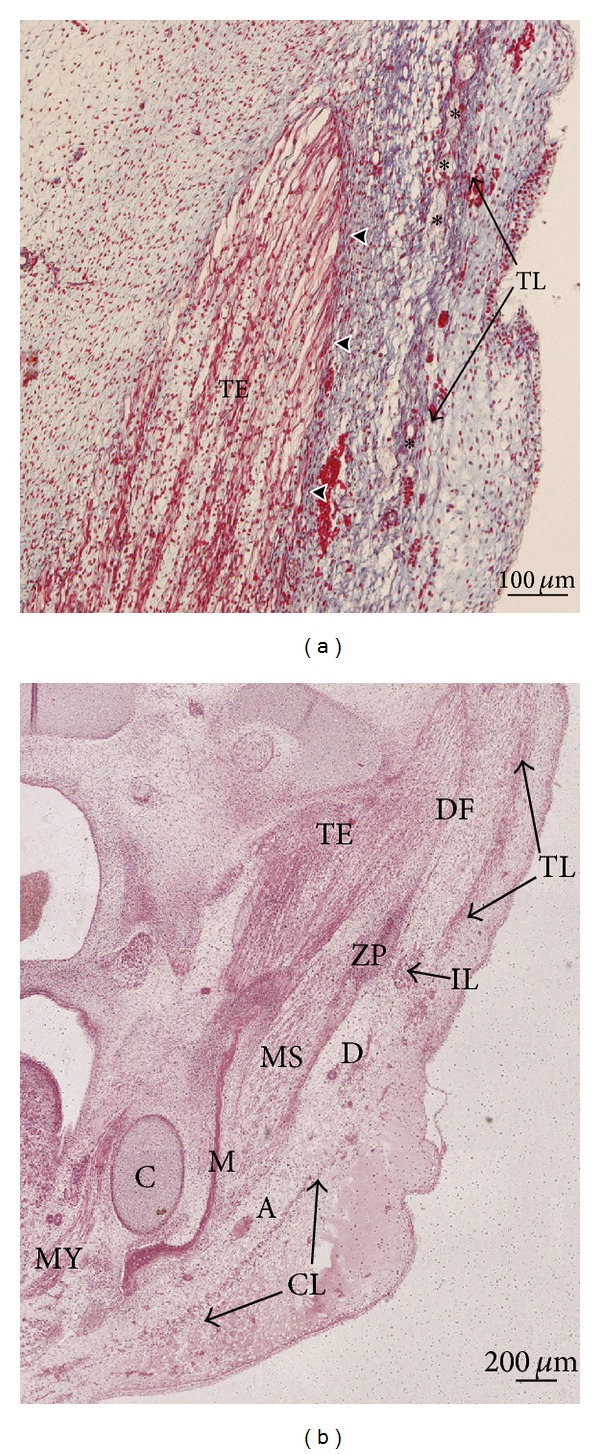
(a) Human embryo BR4 (28 mm GL; 8 weeks of development). Frontal section. Haematoxylin-eosin staining. The anlage of the deep layer of the temporal fascia is visible (arrowheads). Asterisk: temporal branches of the facial nerve. Bar: 100 *μ*m. (b) Human embryo Br4 (28 mm GL; 8 weeks of development). Frontal section. Haematoxylin-eosin staining. The infraorbital lamina (IL) is continuous with the temporal lamina (TL). Bar: 200 *μ*m.

In the temporal region, the anlage of the temporalis muscle and the deep layer of temporal fascia are observed. The superficial layer of the temporal fascia contains the temporal branches of the facial nerve and is separated from the anlage of the deep layer of the temporal fascia by lax mesenchyme (Figure 3(a)). The superficial layer of the temporal fascia derives from the temporal lamina. The temporal and the infraorbital lamina are continuous (Figure 3(b)).

**Figure 4:**
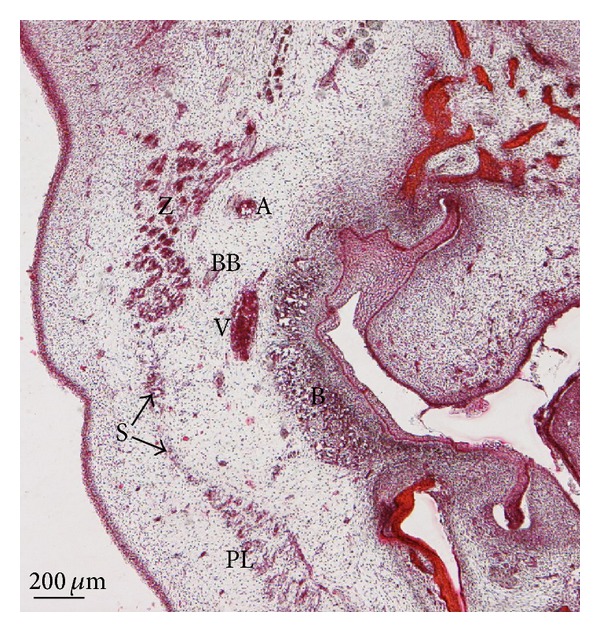
Human fetus B606 (40 mm GL; 9 weeks of development). Frontal section. Haematoxylin-eosin staining. The platysma muscle (PL) is continuous with the superficial musculoaponeurotic system (S). Bar: 200 *μ*m.

During the 9th-10th weeks, the platysma muscle arises from the cervical lamina and its mandibular extension. The platysma muscle is continuous with a fibromuscular lamina, thin and discontinuous, arranged on the same plane as the zygomaticus major muscle. The SMAS originates from this lamina while the zygomaticus major muscle originates from the infraorbital lamina. The anlage of the SMAS encloses the facial vessels and the buccal branches of the facial nerve (Figure 4).

**Figure 5:**
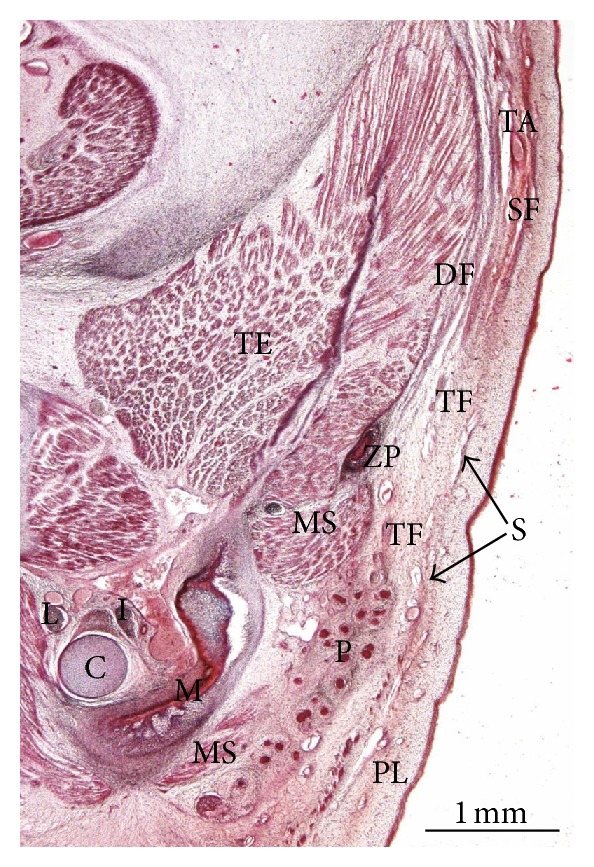
Human fetus B503 (48 mm GL; 10 weeks of development). Frontal section. Haematoxylin-eosin staining. The anlage of superficial musculoaponeurotic system (S) is not continuous with the anlage of the superficial layer of the temporal fascia (SF). Bar: 1 mm.

The superficial layer of the temporal fascia contains the temporal superficial artery. The anlage of the SMAS is arranged on the same plane as the anlage of the superficial layer of the temporal fascia, but there appears to be no continuity between both of them (Figure 5).

**Figure 6:**
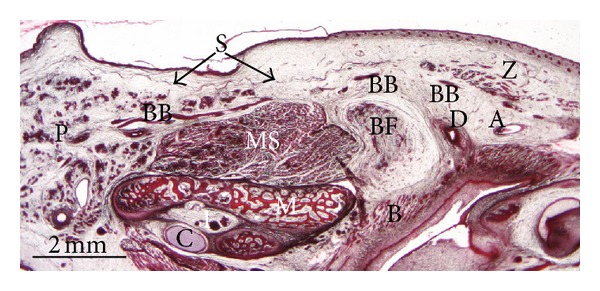
Human fetus ST8 (105 mm GL; 14 weeks of development). Transversal section. Haematoxylin-eosin staining. The superficial musculoaponeurotic system (S) encloses the buccal branches of the facial nerve (BB). Bar: 2 mm.

**Figure 7:**
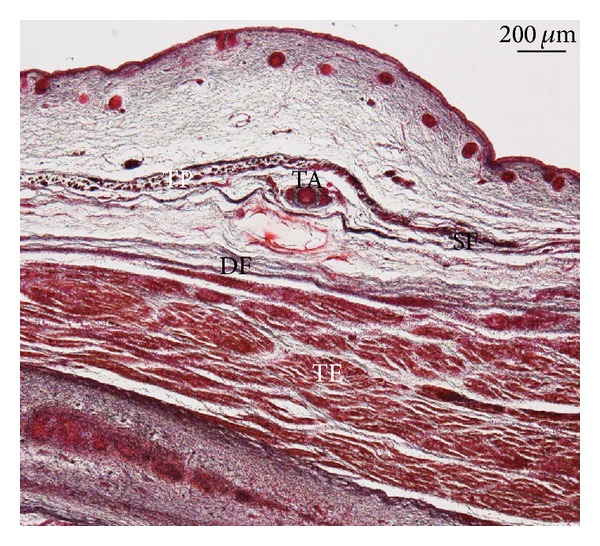
Human fetus ST8 (105 mm GL; 14 weeks of development). Transversal section. Hematoxylin-eosin staining. The temporoparietalis muscle (TP) is visible. Bar: 200 *μ*m.

In specimens at 13th-14th weeks of development, the anlage of buccal fat pad and its capsule can be observed in relation to the masseter and buccinators muscles and the parotic duct enclosed by the SMAS (Figure 6). In two specimens, we observed bilaterally that the superficial layer of the temporal fascia contained the temporoparietalis muscle (Figure 7).

**Figure 8:**
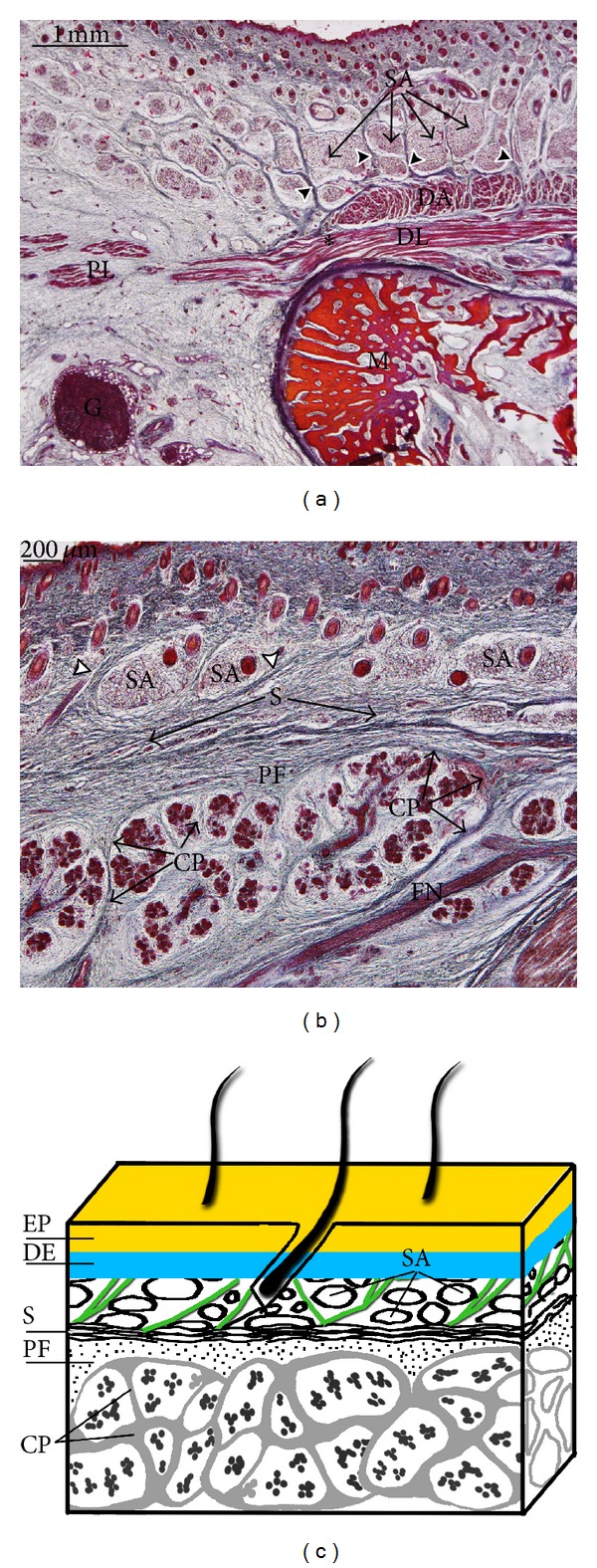
(a) Human fetus B28 (150 mm GL; 17 weeks of development). Transversal section. Haematoxylin-eosin staining. Some fibres (asterisk) of the platysma muscle (PL) are continuous with the depressor labii inferioris (DL). Arrowhead: fibrous septa. Bar: 1 mm. (b) Human fetus B28 (150 mm GL; 17 weeks of development). Transversal section. Haematoxylin-eosin staining. The superficial musculoaponeurotic system (S) encloses anlage of the parotid fascia (PF). Arrowheads: fibrous septa. Bar: 200 *μ*m. (c) Schematic diagram of the arrangement of the superficial musculoaponeurotic system (S) and the anlage of the parotid fascia (PF) in specimens at 17 weeks of development. Fibrous septa are in green.

During the 17th week of development, the insertion of the platysma muscle in the mandible and its continuity with the depressor labii inferioris muscle may be observed. Superficially, the depressor anguli oris muscle is observed (Figure 8(a)). In the parotid region, the platysma muscle and the SMAS are separated from the parotid gland capsule propia by connective tissue, the anlage of the parotid fascia. Superficial to the platysma muscle and the SMAS, the anlage of the superficial adipose layer with fibrous septa is observed (Figures 8(b) and 8(c)).

## 4. Discussion

The facial musculature derives from the mesenchyme of the second arch and is innervated by the facial nerve. The premuscular facial masses are formed between the 6th and 7th weeks of development. The migration of the differentiating premioblast and early myoblast extends from the region of the second pharyngeal arch in sheet like laminae. The superficial lamina spreads from a location caudal to the external acoustic meatus in all directions forming the temporal, occipital, cervical, and mandibular laminae. The superficial mimetic muscles of the face arise from these laminae [23, 28].

In specimens at 8 weeks of development, we have identified the cervical, mandibular, infraorbital, and temporal laminae and located them on the superficial plane of the face and neck. These laminae of mesenchymal origin will give rise to the superficial mimetic muscles and fasciae (superficial layer of the temporal fascia, epicranial aponeurosis, and the SMAS).

In specimens at 17th week of development, we have observed the continuity of the platysma muscle with the depressor labii inferioris muscle. This arrangement has been described in human adults [3, 29] and in rhesus macaque [30].

### 4.1. The Relationship of the Platysma Muscle and the SMAS

From the viewpoint of anatomical and surgical dissection in adults, the platysma muscle is continuous and on the same plane as the SMAS [31, 32]. The continuity of the platysma muscle with the superficial fascia in the parotid region during the development has been described by Zigiotti et al. [24]. The SMAS has been considered a fibrous degeneration of the platysma muscle [12, 33] or an extension of the cervical superficial fascia [4]. The platysma muscle could be an evolutive form of the panniculus carnosus present in lower animals [34].

In adults, it has been reported that the platysma muscle is subject to considerable variation, sometimes forming a very thin layer, largely interspersed among connective tissue. Its extension on the face may also vary considerably, reaching the zygomatic arch and the eyelid orbicular muscle [29, 35].

**Figure 9:**
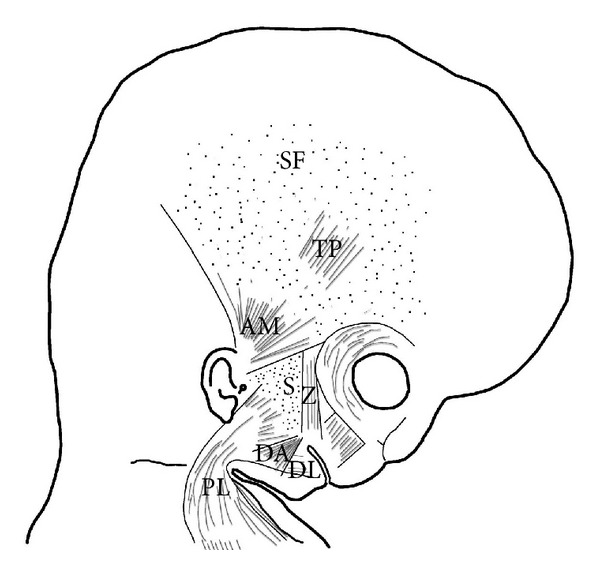
Schematic diagram of the arrangement of the anlage of the superficial musculoaponeurotic system (S). The platysma muscle (PL) derives from the cervical lamina and its mandibular extension. The depressor labii inferioris (DA) and depressor anguli oris (DL) muscles derive from the mandibular lamina. The zygomaticus major muscle (Z) derives from the infraorbital lamina. The superficial layer of the temporal fascia (SF) and the auricularis (AM) and temporoparietalis (TP) muscles originate from the temporal lamina.

Our results coincide with Gasser [23] in that the platysma muscle arises from the cervical lamina and its mandibular extension. Nonetheless, its development is very variable, occupying the lower part of the parotid and cheek regions. The SMAS arises from the upper part of the mandibular extension of the cervical lamina. The anlage of the SMAS is continuous with the platysma muscle (Figure 9).

### 4.2. Parotid Region

Regarding the parotid region in adults, it has been reported that the parotid fascia corresponds to the SMAS [16, 33]. However, other authors have considered that it is possible to distinguish between both structures [4, 5, 12, 14, 15, 18, 19].

Zigiotti et al. [24] studied serialised cross sections of 10 human fetuses and found no structure that might be considered to constitute the parotid fascia. Our findings indicate that the lower part of the parotid region is enclosed by the platysma muscle, while the upper part of the parotid region is enclosed by the anlage of the SMAS. The parotid gland capsule propria arises from the mesenchyme enclosing the glandular acini forming intraglandular septae. In specimens at the 17th week of development, a layer of connective tissue is observed between the capsule propia and the plane of the platysma muscle and SMAS, giving rise to the parotid fascia. Our study does not indicate the presence of an adipose layer between the anlage of the SMAS and that of the parotid fascia.

### 4.3. Labial Region

It has been reported that the SMAS is not identifiable in the labial region [12, 15, 16, 18]. However, some authors describe a specific type of SMAS in the labial region [14]. It has also been reported that the SMAS is clearly visible in the upper lip [11]. We have not observed any layer similar to the SMAS in the labial region as has been described in the parotid and the cheek regions. In adults, some authors affirm that the SMAS finalizes at the nasolabial fold [9]. Macchi et al. [15] have stated that it encloses the zygomaticus muscles. In the specimens examined in this study, we have observed that the anlage of the SMAS is on the same plane and discontinuously reaches the zygomaticus major muscle.

### 4.4. Temporal Region

In adults, some authors have described that the SMAS and the superficial layer of the temporal fascia are on the same plane and are continuous [5, 9, 12, 15, 16, 20–22, 36]. Other authors have considered that no such continuity exists between these structures [3, 18, 19]. Our results indicate that the superficial layer of the temporal fascia and the auricularis muscles originate from the temporal lamina. During the period analyzed, we have observed no continuity between the anlage of the SMAS and that of the superficial layer of the temporal fascia. The temporal branches of the facial nerve and the superficial temporal artery are enclosed by the SMAS and penetrate the anlage of the superficial layer of the temporal fascia. In adults, it has been described that the temporal branches of the facial nerve are arranged in the thickness of the superficial layer of the temporal fascia [4, 37]. This arrangement may have led some authors to consider that, in adults, the superficial layer of the temporal fascia is delaminated [10, 36]. This relationship between the temporal branches of the facial nerve and the superficial layer of the temporal fascia is important in certain aesthetic and plastic surgery techniques [7, 10, 37].

Bilaterally, in two specimens, one at the 12th week and the other at the 13th week of development, the superficial layer of the temporal fascia contained the temporoparietalis muscle. We believe that this muscle also arises from the mesenchyme, forming the temporal lamina. In adults, it has been pointed out that the temporoparietalis muscle is a muscular lamina of variable development located between the frontal belly of the epicranius muscle and the anterior and posterior auricularis muscles [3]. This muscle may correspond to the inconstant orbitoauricularis muscle of the rhesus macaque [30].

## 5. Conclusion

In summary, the superficial mimetic muscles derive from the mesenchyme of the second arch which migrates during development and forms premuscular laminae, related variably among each other, depending on the degree of migration. The SMAS arises from the mesenchyme of the mandibular extension of the cervical lamina enclosing the upper part of the parotid region and the cheek. The anlage of the SMAS is in continuity with the platysma muscle. We have not observed continuity between the anlage of the SMAS and that of the superficial layer of the temporal fascia and the zygomaticus major muscle. In the labial region, we have not observed any structure similar to the SMAS.

## Abbreviations

A: Facial artery
B: Buccinator muscle
BB: Buccal branches of the facial nerve
BF: Anlage of the buccal fat pad
CP: Capsula propria of the parotid gland
C: Meckel's cartilage
CL: Cervical lamina
D: Parotid duct
DA: Depressor anguli oris muscle
DE: Dermis
DF: Deep layer of the temporal fascia
EP: Epidermis
FN: Facial nerve
G: Submandibular node lymph
I: Inferior alveolar nerve
IN: Infraorbital nerve
J: External jugular vein
L: Lingual nerve
M: Mandible
MM: Marginal mandibular branch of the facial nerve
MS: Masseter muscle
MY: Mylohyoid muscle
P: Anlage of the parotid gland
PL: Platysma muscle
SA: Superficial adipose layer
SB: Submandibular gland
SF: Superficial layer of the temporal fascia
ST: Sternocleidomastoid muscle
T: Temporalis muscle
TA: Superficial temporal artery
TE: Temporalis muscle
TF: Temporalis branches of the facial nerve
TL: Temporal lamina
V: Facial vein
Z: Zygomaticus major muscle
ZP: Zygomatic process of the squamous part of the temporal bone.

## References

[1] Mitz V, Peyronie M (1976). The superficial musculo aponeurotic system (SMAS) in the parotid and cheek area. *Plastic and Reconstructive Surgery*.

[2] Federative Committee on Anatomical Terminology (1998). *Terminologia Anatomica*.

[3] Standring S (2008). *Gray's Anatomy: The Anatomical Basis of Clinical Practice*.

[4] Stuzin JM, Baker TJ, Gordon HL (1992). The relationship of the superficial and deep facial fascias: relevance to rhytidectomy and aging. *Plastic and Reconstructive Surgery*.

[5] Gola R, Cheynet F, Faissal A, Guyot I (1998). Apports récents en Chirurgie Esthétique Faciale (techniques endoscopiques exclues). *Revue de Stomatologie et de Chirurgie Maxillo-Faciale*.

[6] Cesteleyn L, Helman J, King S, Van De Vyvere G (2002). Temporoparietal fascia flaps and superficial musculoaponeurotic system plication in parotid surgery reduces Frey’s syndrome. *Journal of Oral and Maxillofacial Surgery*.

[7] Zani R, Fadul R, Dias Da Rocha MA, Santos RA, Alves MCA, Ferreira LM (2003). Facial nerve in rhytidoplasty: anatomic study of its trajectory in the overlying skin and the most common sites of injury. *Annals of Plastic Surgery*.

[8] Ferreira LM, Hochman B, Locali RF, Rosa-Oliveira LMQ (2006). A stratigraphic approach to the superficial musculoaponeurotic system and its anatomic correlation with the superficial fascia. *Aesthetic Plastic Surgery*.

[9] Gassner HG, Rafii A, Young A, Murakami C, Moe KS, Larrabee WF (2008). Surgical anatomy of the face: implications for modern face-lift techniques. *Archives of Facial Plastic Surgery*.

[10] Trussler AP, Stephan P, Hatef D, Schaverien M, Meade R, Barton FE (2010). The frontal branch of the facial nerve across the zygomatic arch: anatomical relevanceof the high-SMAS technique. *Plastic and Reconstructive Surgery*.

[11] Pensler JM, Ward JW, Parry SW (1985). The superficial musculoaponeurotic system in the upper lip: an anatomic study in cadavers. *Plastic and Reconstructive Surgery*.

[12] Thaller SR, Kim S, Patterson H, Wildman M, Daniller A (1990). The submuscular aponeurotic system (SMAS): a histologic and comparative anatomy evaluation. *Plastic and Reconstructive Surgery*.

[13] Har-Shai Y, Bodner SR, Egozy-Golan D (1997). Viscoelastic properties of the superficial musculoaponeurotic system (SMAS): a microscopic and mechanical study. *Aesthetic Plastic Surgery*.

[14] Ghassemi A, Prescher A, Riediger D, Axer H (2003). Anatomy of the SMAS revisited. *Aesthetic Plastic Surgery*.

[15] Macchi V, Tiengo C, Porzionato A (2009). Histotopographic study of the fibroadipose connective cheek system. *Cells Tissues Organs*.

[16] Delmar H (1994). Anatomy of superficial planes of the face and neck. *Annales de Chirurgie Plastique et Esthetique*.

[17] McKinney P, Gottlieb J (1985). The relationship of the great auricular nerve to the superficial musculoaponeurotic system. *Annals of Plastic Surgery*.

[18] Gardetto A, Dabernig J, Rainer C, Piegger J, Piza-Katzer H, Fritsch H (2003). Does a superficial musculoaponeurotic system exist in the face and neck? An anatomical study by the tissue plastination technique. *Plastic and Reconstructive Surgery*.

[19] Gosain AK, Yousif NJ, Madiedo G (1993). Surgical anatomy of the SMAS: a reinvestigation. *Plastic and Reconstructive Surgery*.

[20] Tellioğlu AT, Tekdemir I, Erdemli EA, Tüccar E, Ulusoy G (2000). Temporoparietal fascia: an anatomic and histologic reinvestigation with new potential clinical applications. *Plastic and Reconstructive Surgery*.

[21] Campiglio GL, Candiani P (1997). Anatomical study on the temporal fascial layers and their relationships with the facial nerve. *Aesthetic Plastic Surgery*.

[22] Accioli de Vasconcellos JJ, Britto JA, Henin D, Vacher C (2003). The fascial planes of the temple and face: an enbloc anatomical study and a plea for consistency. *British Journal of Plastic Surgery*.

[23] Gasser RF (1967). The development of the facial muscles in man. *American Journal of Anatomy*.

[24] Zigiotti GL, Liverani MB, Ghibellini D (1991). The relationship between parotid and superficial fasciae. *Surgical and Radiologic Anatomy*.

[25] O’Rahilly R, Müller F (2001). *Human Embryology and Teratology*.

[26] O’Rahilly R, Müller F (2010). Developmental stages in humans embryos: revised and new measurements. *Cells Tissues Organs*.

[27] McManus JFA, Mowry RW (1968). *Técnica Histológica*.

[28] Sperber GH (2001). *Craniofacial Development*.

[29] Bergman RA, Thompson SA, Afifi A, Saadeh FA (1988). *Compendium of Human Anatomic Variation: Text, Atlas, and World Literature*.

[30] Burrows AM, Waller BM, Parr LA (2009). Facial musculature in the rhesus macaque (Macaca mulatta): evolutionary and functional contexts with comparisons to chimpanzees and humans. *Journal of Anatomy*.

[31] de Castro CC (1991). Superficial musculoaponeurotic system-platysma: a continuous study. *Annals of Plastic Surgery*.

[32] Cárdenas-Camarena L, González LE (1999). Multiple, combined plications of the SMAS-platysma complex: breaking down the face-aging vectors. *Plastic and Reconstructive Surgery*.

[33] Jost G, Levet Y (1984). Parotid fascia and face lifting: a critical evaluation of the SMAS concept. *Plastic and Reconstructive Surgery*.

[34] Bela Fodor P (1993). Editorial: from the panniculus carnosus (PC) to the superficial fascia System (SFS). *Aesthetic Plastic Surgery*.

[35] Bryce TH, Sharpey E, Symimgton J, Bryce TH (1923). Myology. *Quain's Elements of Anatomy*.

[36] Agarwal CA, Mendenhall SD, Foreman KB, Owsley JQ (2010). The course of the frontal branch of the facial nerve in relation to fascial planes: an anatomic study. *Plastic and Reconstructive Surgery*.

[37] Serra-Renom JM, Vila-Rovira R (1995). *Endoscopia en cirugía plástica estética*.

